# Microencapsulation for the Therapeutic Delivery of Drugs, Live Mammalian and Bacterial Cells, and Other Biopharmaceutics: Current Status and Future Directions

**DOI:** 10.1155/2013/103527

**Published:** 2012-12-04

**Authors:** Catherine Tomaro-Duchesneau, Shyamali Saha, Meenakshi Malhotra, Imen Kahouli, Satya Prakash

**Affiliations:** ^1^Biomedical Technology and Cell Therapy Research Laboratory, Departments of Biomedical Engineering and Physiology and Artificial Cells and Organs Research Center, Faculty of Medicine, McGill University, 3775 University Street, Montreal, QC, Canada H3A 2B4; ^2^Faculty of Dentistry, McGill University, 3775 University Street, Montreal, QC, Canada H3A 2B4; ^3^Department of Experimental Medicine, McGill University, 3775 University Street, Montreal, QC, Canada H3A 2B4

## Abstract

Microencapsulation is a technology that has shown significant promise in biotherapeutics, and other applications. It has been proven useful in the immobilization of drugs, live mammalian and bacterial cells and other cells, and other biopharmaceutics molecules, as it can provide material structuration, protection of the enclosed product, and controlled release of the encapsulated contents, all of which can ensure efficient and safe therapeutic effects. This paper is a comprehensive review of microencapsulation and its latest developments in the field. It provides a comprehensive overview of the technology and primary goals of microencapsulation and discusses various processes and techniques involved in microencapsulation including physical, chemical, physicochemical, and other methods involved. It also summarizes the state-of-the-art successes of microencapsulation, specifically with regard to the encapsulation of microorganisms, mammalian cells, drugs, and other biopharmaceutics in various diseases. The limitations and future directions of microencapsulation technologies are also discussed.

## 1. Introduction

Microencapsulation has gained importance in the fields of cell and tissue engineering, as well as in the development of drug formulations and oral delivery systems. There are a number of already marketed microencapsulated products for the delivery of pharmaceutics [1]. The term microencapsulation, in this work, encompasses the terms microcapsules, microparticles, microspheres, and microemulsions. Generally, the term microsphere is employed for a homogeneous structure made of one continuous phase, and the term microcapsule is used for a reservoir-like structure with a well-defined core and envelope/coat. There exist a variety of microcapsules which can differ in size, composition, and function. The characteristics of the microcapsules ultimately depend on the final goal of the encapsulated product, as they can be used to entrap all sorts of substances: solids, liquids, drugs, proteins, bacterial cells, stem cells, and so forth. With such a range of substances that can be entrapped, one can conclude that microcapsules can have an assortment of objectives and applications, whether for drug delivery, enzyme retrieval, artificial cell and artificial tissue delivery, and delivery of microorganisms.

This paper provides an up-to-date review of microencapsulation and its latest developments. It provides a comprehensive overview of microencapsulation technology, the primary goals of microencapsulation, and the processes and techniques involved. This includes the physical, chemical, physicochemical, and other methods. Specifically, this paper comprehensively discusses the use of microencapsulated microorganisms in renal diseases, cardiovascular diseases, colorectal cancer, inflammatory bowel disease, and others. Microencapsulation for mammalian cells is described for diabetes, hepatic diseases, parathyroid insufficiency, anemia, cancer, and neurodegenerative diseases. The use of microencapsulated drugs and other pharmaceutics focuses on hormone therapy, gastrointestinal disorders, diabetes, pulmonary diseases, periodontitis, and hypertension. The limitations and future directions of microencapsulation are also discussed.

## 2. Goals of Microencapsulation

**Figure 1:**
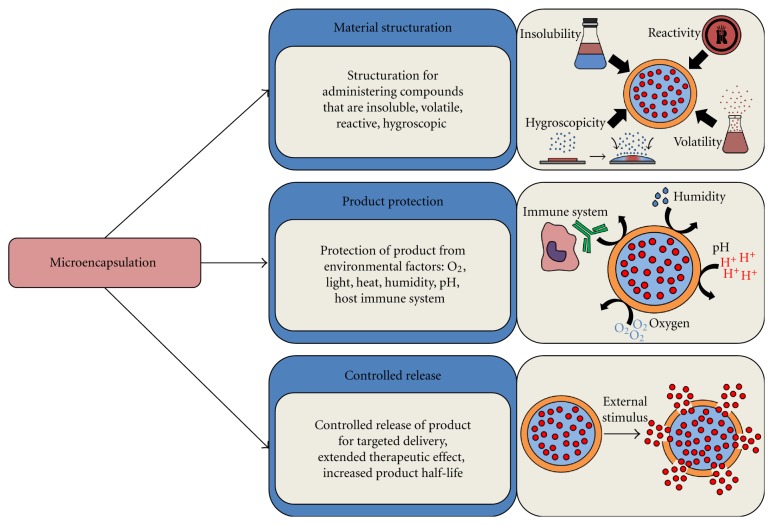
The main biopharmaceutical goals of microencapsulation: microencapsulation can be used to achieve material structuration, therapeutic product protection, and targeted delivery and/or controlled release of the encapsulated biotherapeutics.

Microencapsulation can be used to achieve a number of objectives. Some goals of microencapsulation include material structuration, protection of the enclosed product, and controlled release of the encapsulated contents, as shown in Figure 1. Microcapsules can provide structuration to compounds that are normally difficult to administer due to factors such as the material's insolubility, volatility, reactivity, hygroscopicity, and physical state [93]. Microcapsules may also serve the role of protecting the encapsulated contents to prevent the degradation of the product due to external environmental factors such as oxygen, light, heat, and humidity which could destroy any labile compound. Protection by microcapsules may also be required when orally administering a therapeutic, due to exposure to the harsh conditions of the upper gastrointestinal tract (GIT). In addition, the host's immune system would quickly lead to the implanted cells' rejection and undesired side effects if the cells are recognized as foreign. Immunoprotection and immunoisolation may be achieved by a microcapsule, important for the *in vivo* delivery and implantation of mammalian cells, such as stem cells, for tissue and cell engineering applications. The capability of microcapsules to serve the purpose of immunoprotection has been well demonstrated in a number of disease contexts, including type 1 diabetes, Parkinson's disease, Alzheimer's disease, cancers, and other disorders [48, 94–97]. Microcapsules may also serve to permit the controlled release of the encapsulated contents, which can be regulated by chemical, physical, and mechanical factors. A controlled release can permit a longer and more efficient therapeutic effect of an enzymatic by-product, which, otherwise, may have a limited half-life *in vivo*. It may also regulate the release of the encapsulated product at the desired time, rate, dose, and site of action.

## 3. Microencapsulation Methods

There are a number of techniques that can be used to fabricate microcapsules, depending on the desired characteristics and applications of the final microcapsule formulation. These techniques can be broadly categorised into chemical, physical and physicochemical methods, as highlighted in Table 1. 

### 3.1. Chemical Methods of Microencapsulation

Chemical methods of microencapsulation include solvent evaporation, interfacial cross-linking, interfacial polycondensation, *in situ* polymerization, and matrix polymerization. Solvent evaporation is a technique used by many companies for the production of microcapsules, especially for drug encapsulation, as the method often requires heat [98]. The process necessitates that the core material be dissolved/dispersed in the coating solution followed by agitation in the liquid vehicle to obtain the desired microcapsule size [98]. This mixture is then heated to evaporate the solvent, followed by temperature reduction.

The microencapsulation method of interfacial polycondensation, also termed interfacial condensation polymerization, was pioneered by Chang [99]. The method involves the Schotten-Baumann reaction between an acid chloride and a compound containing an active hydrogen atom [100]. This reaction involves two polymeric reactants in a polycondensation that meet and form thin walls at the microcapsule interface [101]. The method of interfacial cross-linking originated from that of interfacial polycondensation and also involves the Schotten-Baumann reaction. The process involves a bifunctional monomer containing active hydrogen atoms which, during encapsulation, are replaced by a biopolymer, such as a protein [102]. At the interface of the emulsion, the membrane of the microcapsule is formed by the reaction of an acid chloride with the functional groups of the protein. A carbohydrate or starch may also be added for an increased modulation of biodegradability and other physical properties [103].

*In situ* and matrix polymerizations are methods used in a number of microencapsulation processes for coating of the microcapsule. *In situ* polymerization is characterized by the fact that the reactants are not included in the core material, but, rather, polymerize together to form the particle surface [104]. Solidification and stabilization can then be achieved by a number of methods. Matrix polymerization, on the other hand, involves the embedding of the core material in a polymeric matrix during particle formation. This is the case in spray drying, using heat as a physical method of polymerization. In terms of a chemical method of polymerization, this can be achieved using matrices such as epoxy [104].

### 3.2. Physical Methods of Microencapsulation

Physical methods include spray drying, fluid-bed/pan coating, centrifugal extrusion, vibrating nozzle, and spinning disk microencapsulation. For spray drying, an emulsion is prepared by the dispersion of an oil core material or water-soluble active ingredient into a concentrated coating material. The emulsion can then be atomized into a spray of droplets using a rotating disc and a short exposure in a heated compartment to allow water to evaporate, yielding dry microcapsules. Spray drying is an economical method that can allow for the encapsulation of labile materials such as proteins and microorganisms [21, 105].

Fluid-bed coating is a microencapsulation technique used extensively to encapsulate pharmaceuticals into coated particles or tablets [106]. It is a variation of the pan coating method, one of the oldest industrial procedures, where solid particles are mixed with a dry coating material that is heated to surround the particle cores [107]. Solid particles (or liquids absorbed into porous solids) are suspended on a jet of air followed by the application of a coating material using a liquid spray. The resulting shells are solidified by cooling or solvent vaporization, and the process is repeated until the microcapsule walls are of the desired thickness. The Wurster fluid bed system, where the spray nozzle is located below the particle fluidized bed, is one commonly used system for this type of particle coating [108].

Centrifugal extrusion is an easy-to-scale-up microencapsulation technique that involves the use of a spinning extrusion head made up of concentric nozzles [109]. The microcapsule core and coating materials, both immiscible with each other, are pushed through the concentric nozzles forming a flow that splits into droplets following clearing of the nozzle. Depending on the materials used during microencapsulation, solidification of the droplets can then be undertaken by cooling or gelation methods.

Vibrating nozzle, also termed vibrating-jet, is a popular microencapsulation method [110, 111]. The liquid material to be encapsulated is extruded through a nozzle at a specific flow rate, forming a laminar jet. Unlike the centrifugal extrusion technique which relies on natural and irregular disturbances leading to irregular size and shape, this method uses a permanent sinusoidal force at determined frequencies, forming microcapsules of uniform distribution [112]. As in centrifugal extrusion, the solidification of the microcapsules can be undertaken using cooling or gelation.

Spinning disk, also termed rotational suspension separation, is another physical technique for microencapsulation. A mixture is formed with the material for the internal core of the microcapsule and the liquid microcapsule coating material. This dispersion is then flowed onto a turning disk, causing the microcapsules (and coating material shells) to be thrown off of the rim of the disk followed by solidification using cooling or gelation techniques. Purification is then performed to isolate the microcapsules from coating material particles.

### 3.3. Physicochemical Methods of Microencapsulation

Physicochemical encapsulation techniques involve ionotropic gelation, polyelectrolyte complexation, coacervation, and supercritical fluid technology. Ionotropic gelation relies on the ability of polyelectrolytes to cross-link when in the presence of counterions, leading to their gelation [113]. This process has been extensively studied using natural polyelectrolytes such as alginate [114, 115], chitosan [116], carboxymethyl cellulose [117], and gellan gum [118]. Gelated beads are produced by the addition of polymeric drops, containing the anionic therapeutic to be encapsulated, into an aqueous solution of polyvalent cations [113]. Ionic cross-linking forms a three-dimensional lattice due to the diffusion of cations into the polymeric drops. Polyelectrolyte complexation, the addition of polycations or polyelectrolytes to the surface of the beads, can be used to further improve the mechanical strength and permeability of the gelated beads.

Coacervation, also termed phase separation, is one of the oldest and most widely used methods of encapsulation that relies on polymer-polymer incompatibility [119]. This technique can be classified into simple and complex coacervation. Simple coacervation involves the addition of a salt or alcohol into the polymeric mixture, promoting the liquid-liquid phase separation and the formation of coacervate polymer droplets [120]. Complex coacervation occurs in the presence of two phases and modification of the aqueous phase pH. This leads to the formation of a membrane at the interface of the coacervate polymer droplets. Subsequently, the membrane can be solidified and further stabilized by polymer cross-linking.

Supercritical fluid technology has recently gained pharmaceutical interest for the formation of particles that are monodispersed with the capability to form nanosized particles [121, 122]. Supercritical fluids can form particles by rapid depressurization or by exceeding the saturation point of a solute by dilution, as well as a combination of both of these processes [122]. Because these reactions occur quicker in supercritical fluids, as compared to liquids, nucleation and spinodal decomposition over crystal growth are promoted, leading to the generation of small particles.

In summary, microencapsulation has strong therapeutic potential, and using the previously methods, microcapsules can be suitably designed for a specific purpose. The following section discusses the use of microencapsulation for the delivery of microorganisms, mammalian cells and drugs, and other pharmaceutics. The following section comprehensively discusses the disease applications where microencapsulation can be used.

## 4. Microencapsulated Microorganisms

Microencapsulation has been widely used for the encapsulation and immobilization of microorganisms [114]. Bacterial cell encapsulation is a process that can occur naturally as bacteria proliferate and produce exopolysaccharides, high-molecular-weight polymers composed of sugar residues. The exopolysaccharide structure can act as a protective capsule and reduce the permeability and bacterial exposure to potential adverse environmental factors. Early research used microencapsulation for the immobilization of bacterial cells in the food and dairy industry, as discussed in other reviews [123, 124]. In recent years, the microencapsulation of probiotic cells, “live microorganisms, which, when administered in adequate amounts, confer a health benefit on the host,” has gained interest for the treatment of a number of gastrointestinal and other health disorders [125]. However, orally delivered probiotic cells must be delivered and remain viable through the harsh conditions of the upper GIT. Hence, microencapsulation can be used as a protection for the delivery of the cells. Research focused on microencapsulation of probiotics has proven successful in the contexts of renal failure, cardiovascular diseases, and in colon disorders, as described later.

### 4.1. Microencapsulated Microbes in Renal Failure

Early research in the field of microencapsulated microorganisms was undertaken, by Prakash and Chang, using a genetically modified *Escherichia coli* strain (DH5) containing a urease gene from *Klebsiella aerogenes *[2, 126]. The encapsulation was performed by gelation of alginate in calcium chloride, followed by coating steps with polylysine and alginate, to give rise to alginate-polylysine-alginate (APA) microcapsules containing *E. coli* cells. When administered orally to uremic rats, the encapsulated *E. coli* successfully lowered the levels of plasma urea and ammonia back to normal levels, as well as modulating many markers of renal failure [3]. This was the first report that recorded the use of polymeric membrane artificial cells for the oral delivery of genetically engineered organisms [3]. This research also highlighted microencapsulation as a method to isolate the delivered microorganisms through the GIT transit until excretion, eliminating safety issues associated with the delivery of microorganisms. Research was also undertaken, *in vitro*, with the same *E. coli *but using polyvinyl alcohol microcapsules which have a significantly higher mechanical strength than APA microcapsules [4, 127]. Supplemental research was also performed with *Lactobacillus delbrueckii* capable of removing urea, to respond to concerns of toxicity associated with the use of the genetically engineered *E. coli* strain [8].

 Research by Prakash et al. provided the first research investigating the use of microencapsulated yeast cells, *Saccharomyces cerevisiae*, in renal failure [5]. The research group investigated the oral administration of live yeast cells in APA microcapsules in a renal failure uremic rat model [5]. The study demonstrated that the microencapsulated yeast cells were retained in the microcapsules through the GIT transit but allowed urea to diffuse through the semipermeable membrane of the microcapsule and were acted upon by yeast urease [5]. More importantly, a significant 18% decrease was noted for the urea levels during the 8-week treatment period, demonstrating the efficacy of the formulation as a therapeutic for eliminating the elevated levels of metabolites present in renal failure [5].

### 4.2. Microencapsulated Microbes in Hypercholesterolemia and Cardiovascular Diseases

Microencapsulation of bacterial cells has recently gained interest for the treatment and prevention of hypercholesterolemia. Early work by Garofalo et al. investigated the use of *Pseudomonas pictorum *microencapsulated with alginate-polylysine and open pore agar [9]. Microencapsulated *P. pictorum* was shown to have significant cholesterol depletion activity, with the highest activity by the open pore agar microcapsule formulation [9]. Continuing the same type of work, Jones et al. investigated APA microencapsulated genetically modified bile-salt-hydrolase (BSH-) active *Lactobacillus plantarum *80 (pCBH1) for its capability to break down and remove bile acids [10]. This research established the use of BSH-active microencapsulated organisms for lowering blood serum cholesterol. Following this work, Martoni et al. demonstrated that APA microencapsulated naturally BSH-active *Lactobacillus reuteri* can be successfully delivered to the colon and remain enzymatically active, using a simulated human gastrointestinal model [12]. This probiotic formulation can contribute to a significant cholesterol-lowering effect in cardiovascular diseases, by contributing to the deconjugation of bile salts in the intestine [128]. Further research by Jones et al. demonstrated the use of APA microencapsulated BSH-active *L. reuteri *in a human clinical study, administered as a yogurt formulation [13]. The formulation was shown to reduce low-density lipoprotein (LDL) cholesterol, total cholesterol, apolipoprotein B-100 (apoB-100), and non-high-density lipoprotein (HDL) cholesterol in hypercholesterolemic patients more efficiently than traditional probiotic therapy and other cholesterol-lowering ingredients [13].

 Research by Bhathena et al. also investigated the use of APA microencapsulated bacteria, specifically feruloyl esterase (FAE) active *L. fermentum*, to lower triglyceride and cholesterol levels, major risk factors for coronary artery disease. Research was undertaken with regard to the viability and enzymatic activity of microencapsulated FAE-active *L. fermentum* under simulated gastrointestinal conditions [14, 129]. It was demonstrated that, following gastrointestinal exposure, there was a significant 2.5 log difference in viability between the free and microencapsulated *L. fermentum* cells [14]. The presence of a higher probiotic viability and FAE activity resulted in significant reductions in serum total cholesterol, LDL cholesterol and serum triglyceride levels in diet-induced hypercholesterolemic hamsters [11]. Similar studies were also performed with microencapsulated *L. fermentum *for the treatment and prevention of metabolic syndrome [15].

### 4.3. Microencapsulated Lactobacilli in Colon Diseases

Microencapsulated microbes have also gained interest for the modulation of colonic inflammation, specifically with regard to colon cancer, but potentially for other colonic inflammatory disorders, such as inflammatory bowel syndrome (IBS) and inflammatory bowel disease (IBD). Urbanska et al. investigated the antitumorigenic properties of APA microencapsulated *Lactobacillus acidophilus *in Min (multiple intestinal neoplasia) mice that carry a germline *Apc *mutation which spontaneously develop numerous pretumoric intestinal neoplasms [16]. Administration of the probiotic led to a significant reduction in the number of adenomas and gastrointestinal neoplasias in the treated animals, suggesting that the microencapsulated bacteria could have a role in the development of a successful colon cancer therapeutic.

Further research investigated the ability of APA microencapsulated *L. acidophilus* to suppress intestinal inflammation in mice, for potential applications in chronic inflammatory gut diseases such as IBS and IBD [17]. The administration of the microencapsulated formulation led to significant lowering of proinflammatory cytokine levels [17]. Markers linked to colonic epithelial cell survival were also increased by the microencapsulated *L. acidophilus* formulation [17]. Previously mentioned studies, with regard to FAE-active microencapsulated microbes, have shown significant antioxidant properties, which could also prove beneficial for colon inflammatory disorders [14, 130]. Research into microencapsulated microorganisms is demonstrating great potential for the treatment and prevention of a number of health disorders, and they are summarized in Table 2. 

## 5. Microencapsulated Mammalian Cells

Regenerative medicine is a field focused on the replacement of lost tissue and organs. The delivery of mammalian cells has been proposed to promote the regeneration of organs such as the liver, pancreas, heart, and kidney. Unfortunately, the *in vivo *delivery of mammalian cells raises a number of challenges. These include (1) immune rejection by the host, (2) a loss in cell survival due to aggregation and impaired nutrition, (3) impaired cellular function due to inadequate gene expression, (4) a requirement for a large amount of readily available cells, and (5) a shortage of human cell donors [131, 132]. Due to a shortage of human donors, research has turned to nonhuman mammalian cells, but the aforementioned impediments of immune rejection, impaired cellular function, and readily available cells remain present. Microencapsulated cells can provide an alternative approach to resolve the aforementioned obstacles. One of the earliest works in this field was by Bisceglie, in the 1930s, who demonstrated the use of a polymer membrane to encase mouse tumour cells [133]. These were injected in a pig's abdominal cavity and were shown to successfully survive attacks by the host immune system [133]. Since then, a lot of research has been undertaken in this field. This section presents a synopsis of the most significant research with regard to microencapsulation in cell-based therapies, focusing on the applications of diabetes and hepatic disease.

### 5.1. Microencapsulated Pancreatic Cells to Treat Diabetes

Type 1 diabetes is a growing concern, with an escalating rate of disease prevalence [134]. With the present lack of a successful therapeutic [134], the delivery of insulin secreting pancreatic islet cells (PICs) has proven promising for the treatment of type 1 diabetes [135]. Unfortunately, the routine use of immunosuppressive drugs to prevent the rejection of implanted PIC predisposes patients to infections and increases the risk of cancer development in the late posttransplant period [136, 137]. Microencapsulation can act as a barrier, shielding the delivered pancreatic cells from the host's defences, eliminating the need for immunosuppressive drugs. The first study evaluating the morphology and function of encapsulated islet cells was performed by Lim and Sun in 1980 [22]. This research demonstrated that islet cells remained intact morphologically and functionally for 4 months, *in vitro*. The encapsulated cells were shown to secrete insulin when stimulated with glucose. Further investigations by Lim and Sun involved the intraperitoneal transplantation of encapsulated islet cells in streptozotocin-induced diabetic Wistar Lewis rats. The transplanted encapsulated islet cells maintained normoglycemia for 3 weeks [22]. The rats transplanted with nonencapsulated cells had normoglycemia for only 6–8 days, demonstrating the potential of microencapsulation for the treatment of type 1 diabetes.

 Studies have investigated the use of microencapsulated PICs to maintain normoglycemia in diabetic animal models. Kobayashi et al. investigated the therapeutic advantage of using encapsulated PIC versus free PIC in the diabetes animal model, nonobese diabetic mice [25]. PICs were encapsulated in 5% (w/w) agarose hydrogel and injected directly into the peritoneal cavity and the omental pouch, without any immunosuppressive drug administration [25]. The control group was injected with free PICs [25]. Two weeks following transplantation, the control group was diabetic, as confirmed by intraperitoneal glucose tolerance tests and blood glucose levels [25]. It is to be noted that the free PICs were no longer viable at this time [25]. On the other hand, encapsulated PICs were able to maintain normal blood glucose levels for over 100 days following transplantation [25]. Omer et al. demonstrated similar results with encapsulated porcine neonatal pancreatic cell clusters (NPCCs) capable of differentiating into insulin producing cells when transplanted into streptozotocin-induced diabetic B6AF1 male mice [26]. Microcapsules, containing 1-2 NPCCs, were manufactured using highly purified alginate cross-linked by barium chloride [26]. The diabetic mice were intraperitoneally transplanted with 10,000 islet equivalent (IE) encapsulated NPCCs in the test group and the equivalent number of nonencapsulated NPCCs in the control group, with no addition of immunosuppressive therapy [26]. The NPCCs were removed 2, 6 and 20 weeks following transplantation [26]. The control group (nonencapsulated NPCCs) remained hyperglycemic while the test group (encapsulated NPCCs) was normoglycemic until the completion of the trial [26]. The function of the transplanted NPCCs was confirmed by the reoccurrence of hyperglycemia following their removal at weeks 2 and 6 [26]. The functionality of the NPCCs was further demonstrated by an insulin upsurge and an improvement in the ratio of *β* cell area to total cellular area at week 20, confirming the differentiation of NPCCs into *β* cells [26]. Like Kobayashi et al., Omer et al. confirmed that microencapsulation successfully provides the encapsulated islet cells with immune protection, without the need for immunosuppression. Moreover, Omer et al. showed the differentiation of NPCCs into insulin producing *β* cells, providing great therapeutic potential for the treatment of type 1 diabetes.

Clinical studies are few, but research by Tuch et al. investigated the transplantation of barium alginate microcapsules containing human islet cells in four type 1 diabetic patients [28]. This group successfully demonstrated the safety of this method, with little C-peptide detected, normal renal function, little cytokine release, and no major infection detected during the trial [28]. Unfortunately, the research group makes the point that the efficacy of the method needs improvement for the therapy to be used clinically, although a decrease in glycemia was observed [28]. Notably, the retrieval of the microcapsules following 16 weeks demonstrated that the encapsulated cells were no longer viable [28].

With respect to future human studies, there is a significant shortage of human insulin secreting cells and so the proposal for xenotransplantation. Xenotransplantation brings about concerns of host immune rejection, an obstacle that microencapsulation could potentially overcome. Abalovich et al. performed a preclinical study investigating the potential of encapsulated pig islet cells for xenotransplantation. Type 1 diabetic dogs were transplanted with encapsulated PICs and demonstrated a significant reduction (20%–80%) in insulin necessity after transplantation [27]. Moreover, there was an upsurge of plasma insulin following 6–12 months of transplantation, along with a significant decrease in glycosylated hemoglobin. Thus, Abalovich et al. demonstrated that microencapsulation may be used for xenotransplantation of PICs in humans [27]. Elliott et al. evaluated the function of PIC APA microcapsules in a single type 1 diabetic patient [29]. Following the intraperitoneal implantation of 15,000 IE/kg bodyweight, at week 12, insulin requirement levels were decreased by 30% [29]. The recovery of the microcapsules, following 9.5 years indicated that the PICs were still viable and secreting small levels of insulin [29]. The research by Elliott et al. demonstrates the potential long-term survival of microencapsulated xenogeneic PIC transplanted without the need for immunosuppression [29].

The presented research provides optimism for the future of microencapsulated PICs for the treatment of type 1 diabetes. However, there still is a need for continuing research to demonstrate the cell viability, functionality with respect to insulin secretion, and safety associated with the xenotransplantation and allotransplantations of microencapsulated PICs using long-term clinical studies.

### 5.2. Microencapsulated Hepatic Cells to Treat Liver Disease

Hepatic diseases, including acute liver failure, chronic liver disease, and congenital metabolic liver disease, require the restoration of liver function [138]. Orthotropic liver transplantation is currently the only effective treatment for end-stage liver disease [33, 139–142]. However, the shortage of organs, the requirement for immunosuppressive therapy, and the numerous complications associated with liver transplantation limit the overall effectiveness of transplantation [143–145]. Recent studies have investigated liver cell transplant (LCT) as a potential therapeutic but, for effective LCT transplantation, immunosuppression is still a requirement [146]. Microencapsulation has been proposed as a method to address these shortcomings, with some important research presented here. The first study evaluating the therapeutic potential of microencapsulated hepatocytes was performed by Sun et al. [31]. Rat hepatocytes, encased in APA microcapsules, were shown to secrete urea and albumin* in vitro*, two molecules secreted by the normal healthy liver. The encapsulated hepatocytes were transplanted into normal Wistar rats and rats with galactosamine-induced fulminant hepatic failure and still remained viable following 35 days [31]. 

A recent study performed by Teng et al. demonstrated the regeneration of liver cells in BALB/C mice with acute liver failure (ALF) by 70% hepatectomy, using a mixture of microencapsulated rat hepatocytes and human fetal liver stromal cells (FLSCs) supplemented with basal fibroblast growth factor (bFGF) [33]. bFGF was added to increase the metabolic activity of hepatocytes and to promote the self-renewal of human embryonic stem cells [33, 147]. The combined treatment of encapsulated rat hepatocytes, FLSCs, and bFGF enhanced the survival rate by over 86% when compared to the controls with a significant increase in liver mass following 72 hours [33]. Furthermore, immunohistochemical inspections showed decreased levels of necrotic liver cells with increased levels of proliferating liver cells in the periportal areas [33]. This study concluded that a mixture of encapsulated rat hepatocytes and FLSCs supplemented with bFGF improved the survival of mice with ALF, without the requirement for any immunosuppression [33]. The encapsulated cells were also protected from any host immunoreactions, demonstrating the potential for encapsulated hepatocyte xenotransplantation.

### 5.3. Other Applications of Microencapsulated Mammalian Cells

Considerable research has also been performed using the microencapsulation of mammalian cells for other diseases. An important study, performed by Zhang et al., investigated the use of microencapsulated Chinese hamster ovarian (CHO) cells that secrete vascular endothelial growth factor (VEGF) in Sprague-Dawley rats, as a therapeutic for ischemic heart diseases [36]. The encapsulated cells were shown to be protected from immune rejection, with significantly lower levels of anti-CHO in those rats as compared to the ones administered unencapsulated cells [36]. Following three weeks of transplantation, the encapsulated CHO cells were found to be functionally active, secreting VEGF [36]. There was also significant improvement in the cardiac function of the rats treated with encapsulated CHO cells, as demonstrated by a decline in fractional shortening and left ventricular enlargement [36]. This research demonstrates great potential for the use of xenotransplantation for the treatment of ischemic heart disease.

Considerable success has been achieved with microencapsulation in the treatment of other conditions such as severe anemia and neurodegenerative disease and has also found use in parathyroid replacement therapies [40, 41, 97]. Rinsch et al. demonstrated the increase in haematocrit value with immunosuppression for 8 weeks, with the use of encapsulated myoblasts [41]. Likewise, Régulier et al. demonstrated the ability of encapsulated myoblasts to secrete erythropoietin which increased the haematocrit value to over 85% in anaemic mice for 80 days [42]. Wikström et al. observed viable human retinal pigment epithelial in microcapsules for over 3 months, demonstrating the potential of encapsulation to maintain the viability and functionality of the encased cells [97]. Hasse et al. demonstrated the reduction of daily intake of calcium and vitamin D by half, in patients suffering from hypocalcaemia by the administration of encapsulated parathyroid tissue particles [40]. Other interesting research demonstrated that microencapsulated retinal pigment epithelial cells can be beneficial for neurodegenerative diseases like Parkinson's [148]. Genetically engineered cells have shown great potential for the development of a cancer therapy, with microencapsulation allowing to bypass the issue of immune rejection [149].  Table 3 provides a comprehensive list of studies where microencapsulated mammalian cells have been used for therapeutic applications.

## 6. Microencapsulated Drugs and Other Pharmaceutics

Microencapsulation technology has greatly enhanced pharmaceutics research in terms of drug delivery devices. This interdisciplinary field comprising polymer science and emulsion technology has not only covered encapsulation of drugs but also of peptides, proteins, and DNA/RNA therapeutic molecules for controlled release studies. This, in turn, has improved the therapeutic efficacy of the molecule with advantages of low dosage requirements and with the ability to be delivered at the targeted site without enzyme degradation by biological fluid proteins. The controlled release of the drug through microspheres/microcapsules occurs via four known mechanisms, namely, diffusion, dissolution, osmosis, and erosion. The phenomenon of sustained controlled release not only protects the drug from degradation but also protects the body from potential toxic effects of the drug [107]. Most of the commonly used polymers for drug applications are poly (lactic acid) (PLA) and poly (lactic-co-glycolic acid) (PLGA) [90, 150]. Other polysaccharides such as chitosan [80, 151, 152], alginate [153], and lipids [70, 154, 155] have also been explored. The choice of polymer, solvents, stabilizers, and surfactants and the rigidity, integrity, and degradability are important parameters governing the formation of microparticles [98, 156]. As aforementioned, solvent evaporation is accepted by many pharmaceutical industries, and has been used in products already on the market [157]. The microencapsulation methods used also depend on the hydrophilic or hydrophobic properties of the drug or molecule to be encapsulated. 

### 6.1. Microencapsulation of Biological Agents, Food Supplements, Enzymes, and Antibiotics

Biodegradable and biocompatible polymers, such as PLGA, have been used to encapsulate biologically active agents such as risperidone (antipsychotic) [59] and testosterone [60, 158] to form microparticles. Microencapsulation methods have also been developed to deliver an adjuvant or an antigen, encapsulated in PLGA microparticles, as vaccine formulations used for immunization purposes [61, 159]. Likewise, encapsulation of food supplements, such as vitamins and oil substances, has also been performed using emulsion technology [160]. A study, performed by Ratnakar Tandale in 2007, demonstrated the microencapsulation of vitamin C and gallic acid, as model antioxidants, in whey protein [91]. These antioxidants were encapsulated using spray drying and freeze drying methods. The study determined the highest encapsulation ratio of vitamin C: whey protein :  gallic acid, and the formulations were optimized for various storage conditions, such as humidity, temperature, and UV light and dark conditions. [61]. Therapeutic agents such as *β*-agonists, anticholinergics, mucolytics, and antimicrobials have also been proposed to be encapsulated for sustained respiratory drug-delivery applications [159]. Haghpanah et al. demonstrated the use of albumin microparticles to deliver salbutamol sulphate as a model drug. This study compared spray drying versus emulsification method of microencapsulation to achieve highest encapsulation efficiency and determined that spray drying achieved 40% to 60% of encapsulation efficiency in comparison to emulsification, which yielded 1% to 2% encapsulation efficiency [72]. Babtsov et al., demonstrated microencapsulation of protein loaded chitosan nanoparticles by spray drying for pulmonary delivery of drugs [160]. They characterized the microparticles for size and aerodynamic properties. The results showed a protein loading efficiency of 65% to 80% with its release of 75% to 80% from nanoparticles within 15 minutes. The study also characterized the recovered nanoparticles from microspheres for size and zeta potential and found no change in the values [73].

Other site-specific drug-delivery applications of microencapsulation include the administration of an ester prodrug, tazarotene subconjunctivally or periocularly [161]. In addition, a silica-lipid hybrid (SLH) microcapsule was recently developed by Tan et al. for the oral delivery of poorly water soluble drugs [162]. The SLH microcapsules were shown to provide physicochemical and biopharmaceutical advantages as compared to the unmodified drug, celecoxib, and the commercial Celebrex product [162]. The SLH microcapsules are hypothesized to improve celecoxib absorption due to dissolution enhancement [162]. Antibiotics such as microcycline HCl have also been administered locally in the periodontal pocket, to modulate inflammation of the periodontium, achieved by embedding the antibiotic-containing microspheres in a strip made of pectin polysaccharide [76, 86]. Another study, by Zheng et al., demonstrated the successful oral delivery of clarithromycin in chitosan-alginate-ethylcellulose microspheres for the treatment of peptic ulcers caused by *Helicobacter pylori *[68]. This study explored the biodegradable properties of alginate and chitosan, especially the mucoadhesive property of chitosan, which facilitates the absorption of the bioactive agent across the intestinal mucosa.

### 6.2. Microencapsulation of Anticancer Drugs and Genes

Microencapsulation has also been used to deliver anticancer agents. A recent study, by Patel et al., demonstrated the advantage of ionotropic gelation to encapsulate a drug, verapamil HCl in a blend of sodium alginate, hydroxypropyl methylcellulose and hydroxymethylcellulose polymers. The microspheres were characterized for the loading and release kinetics of the drug [77]. Other bioactive molecules such as proteins and DNA/RNA, that are more prone to denaturation, have also been encapsulated by solvent exchange. Likewise, DNA has been encapsulated under reduced shear, to maintain its integrity, for oral delivery applications [62, 82, 163].

Microcapsules have also been developed from biocompatible but nonbiodegradable synthetic polymers. These can be customized according to the application, to prolong the stability and controlled release of the drug. In view of this, microcapsules have been developed using ethyl cellulose by phase separation to encapsulate highly hydrophilic drugs such as nonsteroidal anti-inflammatory drugs (NSAIDs) and diclofenac sodium, used for the treatment of rheumatoid arthritis and other diseases [78]. A study conducted by Khamanga et al. investigated the use of Eudragit RS100 (ERS) and Eudragit RL 100 (ERL) for microsphere preparation via solvent evaporation, encapsulating losartan potassium as a model drug [79]. The study evaluated the effect of polymer concentration and its type on the amount of drug released. This strategy was followed to encapsulate drugs that impose a delivery challenge due to their low-molecular weight and high hydrophilicity.

### 6.3. Microencapsulation of Proteins and Hormones

Apart from drug encapsulation and release kinetic studies, microencapsulation technology has also enabled the oral delivery of high-molecular-weight proteins. Due to their high-molecular weight, these molecules are poorly absorbed by the blood stream and are also sensitive to degradation by the acidic environment of the GIT. The sustained release of peptides, proteins, and hormones, such as leutinizing hormone releasing hormone (LHRH) [164–166], recombinant human growth hormone [105], and calcitonin [65] encapsulated in PLGA microcapsules, has been investigated. A study, by Deluca et al., demonstrated the encapsulation of insulin using a blend of acryloyl hydroxyethyl starch (AcHES) hydrogel microparticles with PLGA, an interesting alternative to subcutaneous injection for the management of type 1 diabetes [71]. Another study, involving the encapsulation of insulin, was demonstrated by Caliceti et al. They utilized microspheres prepared using an emulsion technique, comprising a blend of poly(ethylene glycol) with PLA homopolymer and PLG copolymer for a 28-day sustained insulin delivery [167]. Apart from using polymers for microencapsulation techniques, lipids have also been used to encapsulate protein, involving supercritical fluid technology. A study demonstrated the use of this technology using Dynasan 114 and Gelucire 50-02, for the encapsulation of a model protein, bovine serum albumin [87]. All of the previously mentioned applications provided a high therapeutic loading efficiency into the microparticles and provided a sustained release of the active agents [84, 92].

As per the literature, microencapsulation techniques, such as spray drying, phase separation, and emulsion techniques, have been extensively used for the encapsulation of drugs and proteins. However, these techniques pose a limitation in pharmaceutical applications, where the therapeutic molecule may degrade due to thermal and chemical exposure involved during the encapsulation process. Moreover, the presence of solvent residues and the polydispersity of the microcapsules account for undesired toxic effects and a lack of optimal loading of the therapeutic. Thus, alternative methods for encapsulation are being explored to avoid such limitations. One such study, proposed by Li et al., introduced the use of a high-voltage electrostatic field to encapsulate bovine serum albumin (BSA), a model protein, in sodium alginate microcapsules of <100 *μ*m in size with 80 hours of controlled protein release [88]. This study brought a new dimension for the microencapsulation of protein and peptide-based pharmaceutics.

### 6.4. Microencapsulation of Nanohybrid Materials

Other applications of microencapsulation involve the encapsulation of nanoparticles, which can offer great advantages in biomedical applications. For example, the microencapsulation of metals with antioxidative effects can be protected from cellular internalization while maintaining their surface-dependent biomimetic properties. In addition, microencapsulation can prevent an initial burst release of therapeutic drugs, an important limitation of many nanoparticle formulations. Hence, microencapsulation of nanoparticles complexing therapeutic molecules has provided an approach for a controlled drug release [74, 85]. A study by Hasan et al. elaborated the use of ibuprofen and triptorelin acetate as the model lipophilic and hydrophilic drugs encapsulated in poly-*ε*-caprolactone (PCL) nanoparticles, entrapped in microparticles made of ethyl cellulose and ERS. This study revealed a significant reduction in the diffusion of drugs across the double-membrane polymeric wall, providing a longer and more controlled therapeutic release [66]. Microencapsulation of nanoparticles can also serve to reduce cytoxicity effects [168]. A recent study, by Li et al., investigated the microencapsulation of nanoemulsions by spray drying, in which Vitamin E acetate was used as a model lipophilic molecule. This technique helped avoid the instability, the aggregation, and the hydrolysis of nanoparticles in suspension [169]. Likewise, a study performed by Lee et al. demonstrated that cationic lipid nanoparticles encapsulating lipophilic drugs could be microencapsulated in an anionic polymeric membrane, forming microcapsules via ionic interactions. This method produced pH-sensitive microbeads, which proved beneficial for the oral delivery of lipophilic drugs [170]. Microencapsulation of polymeric nanoparticles made of chitosan has also been explored for biomedical applications of pulmonary diseases due to their excellent absorption through mucosal surfaces. Protein-loaded lipid/chitosan nanoparticles were encapsulated in microspheres by spray drying, using mannitol as an aerosol excipient [73, 75]. The produced microspheres showed optimal properties for us in pulmonary diseases. Carbon nanoparticles have also been widely used in biomedical research as a therapeutic vehicle for drugs and genes, specifically with respect to anticancer therapies [171, 172]. A recent study, by Kulamarva et al., investigated the microencapsulation of carbon nanotubes in APA microcapsules for potential oral delivery [89]. The microcapsule membrane has shown resistance against the varying pH of the gastrointestinal system, thereby protecting the encapsulated therapeutic payload, following a controlled release system and releasing the therapeutic at a targeted location.

It is clear from the work summarized in this paper, that microencapsulation is showing great potential for the delivery of a number of drugs and molecular pharmaceutics.  Table 4 summarizes disorders where microencapsulated therapeutic drugs and other agents for the prevention and treatment of health disorders have been investigated. 

## 7. Challenges and Future Outlooks

As described in this paper, microencapsulation is a biomedical technology with remarkable therapeutic potential for a wide range of diseases. The process of microencapsulation can be used in designing therapeutic formulations of microbial cells, mammalian cells, drugs, and other molecular pharmaceutics. In addition to the presented applications, other applications are also promising [173–175]. Given the importance of microencapsulation in various disease applications the technology needs to be further enhanced. One aspect that seems critical is targeted delivery using triggered release of the encapsulated contents due to external trigger factors [176–179]. Other uses of microencapsulation that seem promising are the use of this technology in developing disease models, such as models of tumors for developing pharmaceutical formulations [180, 181]. 

It is also clear that, for each microcapsule formulation, the types and physical and chemical properties of the microcapsules must be optimized. Optimization may involve a number of variables, including the type of microencapsulation process, the encapsulation materials used, and the therapeutic loading capacity. Keeping these characteristics in mind, it is also evident that the future success of microencapsulation must look at the optimization of the methods behind the fabrication of microcapsules. Specifically, characteristics such as permeability, mechanical stability, cell viability, controlled release, targeted delivery, drug stability, and shelf-life of the product, including larger-scale industrial production in therapeutically acceptable production environments, need to be optimized for each intended application.

## Figures and Tables

**Table 1:** Methods for microencapsulation.

Chemical methods	
Solvent evaporation	
Interfacial cross-linking	
Interfacial polycondensation/interfacial condensation polymerization	
*In* *situ* polymerization	
Matrix polymerization	

Physical methods	

Spray drying	
Pan coating	
Fluid-bed coating	
Centrifugal extrusion	
Vibrating nozzle/vibrating-jet	
Spinning disk/rotational suspension separation	

Physicochemical methods	

Ionotropic gelation	
Polyelectrolyte complexation	
Phase separation/coacervation (simple and complex)	
Supercritical fluid technology	

**Table 2:** Microencapsulated microorganism formulations for therapeutic applications.

Disease condition	Microcapsule type	Encapsulated cells type	Delivery method and models	Reference(s)
	APA	*E. coli* DH5	*In vitro *	[2]
	APA	*E. coli* DH5	Rat intragastric gavage	[2]
	APA	*E. coli* DH5	Rat intramuscular injection	[3]
Renal diseases	Polyvinyl alcohol	*E. coli* DH5	*In vitro *	[4]
	APA	*Saccharomyces cerevisiae *	Rat intragastric gavage	[5]
	Alginate chitosan alginate	*E. coli* DH5	*In vitro *	[6]
	Alginate chitosan alginate	*E. coli* DH5	Rat douche	[6]
	Alginate chitosan	*Lactobacillus acidophilus *	*In vitro *	[7]
	APA	*Lactobacillus delbrueckii *	*In vitro *	[8]

	Alginate and alginate-polylysine	*Pseudomonas pictorum *	*In vitro *	[9]
	APA	*Lactobacillus plantarum *80 (pCBH1)	*In vitro *	[10]
	APA	*Lactobacillus fermentum *	Hamster intragastric gavage	[11]
Cardiovascular diseases	APA	*Bifidobacterium longum *	*In vitro *	[12]
	APA	*Lactobacillus reuteri *	*In vitro *	[12]
	APA	*Lactobacillus reuteri *	Human, incorporated in yogurt	[13]
	APA	*Lactobacillus fermentum *	*In vitro *	[14]
	APA	*Lactobacillus fermentum *	Hamster intragastric gavage	[15]

Colorectal cancer	APA	*Lactobacillus acidophilus *	Mouse intragastric gavage	[16]
	Alginate-chitosan	*Lactobacillus acidophilus *	*In vitro *	[7]

Inflammatory bowel syndrome/ inflammatory bowel disease	APA	*Lactobacillus acidophilus *	Mouse intragastric gavage	[17]

	Alginate	*Bifidobacterium longum *	*In vitro *	[18]
		*Bifidobacterium lactis *		
	Alginate	*Bifidobacterium longum *	*In vitro *	[19]
Others		*Bifidobacterium longum *		
	Gelatin	*Bifidobacterium bifidum *	Rat intragastric gavage	[20]
		*Bifidobacterium adolescentis *		
	Reconstituted skim milk with prebiotics	*Bifidobacterium *BB-12	*In vitro *	[21]

**Table 3:** Microencapsulated mammalian cell formulations for therapeutic applications.

Disease condition	Microcapsule type	Encapsulated cells type	Delivery method and models	Reference(s)
Type 1 diabetes mellitus	Alginate polylysine	Rat PICs	Rat intraperitoneal transplant	[22]
	APA	Rat PICs	Rat intraperitoneal transplant	[23]
	APA	Rat PICs	*In vitro *	[24]
			Mouse intraperitoneal transplant	
	Agarose	Mouse PICs	Directly onto the mouse omentum	[25]
	Barium alginate	Porcine NPCCs	Mouse intraperitoneal transplant	[26]
	APA	Porcine PICs	Canine abdominal transplant	[27]
	Barium alginate	Human PICs	Human intraperitoneal transplant	[28]
	APA	Porcine PICs	Human intraperitoneal transplant	[29]
	Alginate poly-L-ornithine	Human PICs	Human intraperitoneal transplant	[30]

Hepatic disease	APA	Rat hepatocytes	Rat intraperitoneal transplant	[31]
	APA	Rat hepatocytes	Mouse intraperitoneal transplant	[32]
	APA	Rat hepatocytes and human FLSCs	Mouse intraperitoneal transplant	[33]
	APA	Porcine hepatocytes	Mouse intraperitoneal transplant	[34]
	APA	Human umbilical cord blood cells	Rat intraperitoneal transplant	[35]

Cardiovascular disease	APA	CHO cells	Rat intramyocardial injection	[36]
	APA	Rat mesenchymal stem cells	Rat intramyocardial injection	[37]

Parathyroid insufficiency	Barium alginate	Human parathyroid tissue	Human forearm and leg transplant	[38]
	Barium alginate	Rat parathyroid tissue	Rat paravertebral muscle transplant	[39]
	Barium alginate	Human parathyroid tissue	Human brachioradial muscle	[40]

Anemia	Polyether-sulfone	Mouse myoblast cells	Mouse dorsal flank transplant	[41]
	Polyether-sulfone	Mouse myoblast cells	Mouse dorsal flank transplant	[42]

	Polyether-sulfone	Human erythroleukemia cells and mouse melanoma cells	Mouse subcutaneous/extraperitoneal transplant	[43]
	APA	Canine kidney cells	Mouse intraperitoneal injection	[44]
	APA	CHO cells	Mouse intraperitoneal transplant	[45]
	APA	CHO cells	Mouse intraperitoneal injection	[46]
	Alginate polylysine	Porcine aortic endothelial cells	Mouse subcutaneous injection	[47]
	APA	Genetically modified hamster kidney cells	Mouse subcutaneous injection	[48]
Cancer	Alginate-polylysine	Human genetically engineered fetal kidney cells	Mouse subcutaneous flank injection	[49]
	APA	Human embryonic genetically engineered kidney cells	Mouse subcutaneous flank injection	[50]
	APA	Mouse myoblasts	Mouse subcutaneous flank injection	[51]
	Alginate	Human fetal genetically engineered kidney cells	Rat intracerebral implantation	[52]
	APA	Mouse genetically engineered myoblasts	Mouse intraperitoneal injection	[53]
	Alginate-polylysine	Porcine aortic endothelial cells	Mouse subcutaneous injection	[54]
	Alginate	Genetically engineered CHO cells	Rat intraperitoneal injection	[55]
	APA	Mouse genetically engineered myeloma cells	Rat subcutaneous injection	[56]

Neurodegenerative diseases	APA	Baby hamster kidney cells	Mouse cerebral cortex implantation	[57]
	Alginate	Neonatal porcine choroid plexus cells	Rat intracranial transplantation	[58]

**Table 4:** Microencapsulated drugs and other biopharmaceutics.

Disease condition	Type of microencapsulation	Encapsulated therapeutic	Delivery method and models	Reference(s)
Psychosis	Poly(vinyl alcohol) and polylactide-co-glycolide	1,2-benzole	Canine intramuscular injection	[59]
		Risperidone		[60]

Vaccines and gene therapy	Poly(lactic-co-glycolic acid)	MN rgp 120, QS 21	Guinea pig subcutaneous injection	[61]
			Mouse intraperitoneal injection	[62]
	Poly(D,L-lactide-co-glycolide)	DNA	Mouse intraperitoneal injection	
			Mouse intragastric gavage	[63]

Hormone therapy	Poly(lactic-co-glycolic acid)	Norethindrone	Baboon injection	
	Poly(lactic-co-glycolic acid)	Estradiol benzoate	Calf base of the ear injection	[60]
	D,L-lactide/glycolic copolymer	Testosterone	*In vitro *	
	Poly(lactic-co-glycolic acid)	Luteinizing hormone releasing hormone	Rat subcutaneous injection	
	Poly lactic acid	Calcitonin	*In vitro *	[64]
		Epidermal growth factor	Rat subcutaneous injection
		Prolactin	
	Poly(lactic-co-glycolic acid)	Calcitonin	*In vitro *	[65]
	Ethylcellulose	Triptorelin acetate poly(caprolactone) nanoparticles	*In vitro *	[66]

Gastrointestinal disorders	Chitosan	Cimetidine	*In vitro *	[67]
	Chitosan-alginate-ethylcellulose	Clarithromycin	Rat intragastric gavage	[68]
	Poly(lactic-co-glycolic acid)	Octreotide	*In vitro *	[65]
	APA	Thalidomide	*In vitro *	[69]

	Glyceryl tripalmitate	Insulin	Mouse subcutaneous injection	[70]
Diabetes	Poly lactic acid		*In vitro *	[64]
	Acryloyl hydroxyethyl starch hydrogel-Poly(lactic-co-glycolic acid)	Insulin	Rat subcutaneous injection	[71]

	Albumin	Salbutamol Sulfate	*In vitro *	[72]
	Lactose-chitosan		*In vitro *	
Pulmonary delivery	Mannitol-chitosan	Insulin		[73]
	Ethyl acetate	Theophylline nanoparticles	*In vitro *	[74]
	Mannitol	Insulin-loaded lipid/chitosan nanoparticles	*In vitro *	[75]

Periodontitis	Poly lactic acid	Minocycline HCl	Canine local administration in periodontal pockets	
		Metronidazole		
		Dibucaine		
	Poly(l-glutamic acid)	Tetracycline HCl		[76]
	Poly(lactic-co-glycolic acid)	Minocycline HCl		
		Flurbiprofen		

	Sodium alginate	Verapamil hydrochloride		
Hypertension	Sodium alginate and hydroxypropyl methylcellulose		*In vitro *	[77]
	Sodium alginate and hydroxypropyl cellulose			
		Losartan potassium	*In vitro *	[78]
	Eudragit RS-Eudragit RL	Verapamil and propranolol	*In vitro *	[79]

	Poly lactic acid	Cisplatin	*In vitro *	
Cancer		Interferon-*α*	Mouse intraperitoneal injection	[64]
	Chitosan	Diphtheria toxoid	Mouse intragastric gavage	[80]
	Alginate-chitosan	Oxaliplatin	Mouse intragastric gavage	[81]

Analgesia and Anesthesia	Ethyl cellulose	Diclofenac sodium	*In vitro *	[82]
	Maltodextrin—whey proteins	Ginger essential oil	*In vitro *	[83]
	Poly(lactic-co-glycolic acid)	Ketoprofen	*In vitro *	
		Lidocaine		[84]
	Poly-*ε*-caprolactone	Diclofenac sodium	*In vitro *	
	Ethylcellulose			[85]
	Ethylcellulose	Ibuprofen poly(caprolactone) nanoparticles	*In vitro *	[66]

	Poly(lactic-co-glycolic acid)	Bovine serum albumin	*In vitro *	[86]
	Poly lactic acid	Tumor necrosis factor	*In vitro *	
		Interleukin-2	Mouse intravenous injection	
		Urokinase	*In vitro *	[64]
		Panmycin	*In vitro *	
	Trimyristin	Bovine serum albumin (BSA)	*In vitro *	
	Gelucire			[87]
	Chitosan			[88]
	APA	Carbon nanotubes	*In vitro *	[89]
	Poly lactic acid	Prednisolone	Rat subcutaneous injection	[90]
	Whey protein concentrate	Ascorbic acid (Vit.C)	*In vitro *	
Other applications		Gallic acid		[91]
	Poly(lactic-co-glycolic acid)	Trenbolone acetate		
		Rgp-120		
		albumin		
		Interferon-*α*	*In vitro *	[60]
	Poly(vinyl alcohol) and polylactide-co-glycolide	Ivermectin		
		Bupivacaine		
	Poly(lactic-co-glycolic acid)	Human growth factor		
	Acryloyl hydroxyethyl starch hydrogel-Poly(lactic-co-glycolic acid)	Insulin	*In vitro *	[71]
		Horseradish peroxidase		
	Glyceryl tripalmitate	Thymocartin	Mouse subcutaneous injection	[70]
	Poly(lactic-co-glycolic acid)	Cyclosporin	*In vitro *	[92]
